# Feasibility study of a randomised controlled trial to investigate the effectiveness of using a humanoid robot to improve the social skills of children with autism spectrum disorder (Kaspar RCT): a study protocol

**DOI:** 10.1136/bmjopen-2017-017376

**Published:** 2017-06-22

**Authors:** Silvana E Mengoni, Karen Irvine, Deepshikha Thakur, Garry Barton, Kerstin Dautenhahn, Karen Guldberg, Ben Robins, David Wellsted, Shivani Sharma

**Affiliations:** 1 Centre for Health Services and Clinical Research, Department of Psychology and Sport Sciences, School of Life and Medical Sciences, University of Hertfordshire, Hatfield, UK; 2 Hertfordshire Community NHS Trust, Welwyn Garden, Hertfordshire, UK; 3 Health Economics Group, Norwich Medical School, University of East Anglia, Norwich, UK; 4 School of Computer Science, University of Hertfordshire, Hatfield, UK; 5 Autism Centre for Education and Research, School of Education, University of Birmingham, Birmingham, UK

**Keywords:** community child health, developmental neurology & neurodisability, child & adolescent psychiatry

## Abstract

**Introduction:**

Interventions using robot-assisted therapy may be beneficial for the social skills development of children with autism spectrum disorder (ASD); however, randomised controlled trials (RCTs) are lacking. The present research aims to assess the feasibility of conducting an RCT evaluating the effectiveness of a social skills intervention using Kinesics and Synchronisation in Personal Assistant Robotics (Kaspar) with children with ASD.

**Methods and analysis:**

Forty children will be recruited. Inclusion criteria are the following: aged 5–10 years, confirmed ASD diagnosis, IQ over 70, English-language comprehension, a carer who can complete questionnaires in English and no current participation in a private social communication intervention. Children will be randomised to receive an intervention with a therapist and Kaspar, or with the therapist only. They will receive two familiarisation sessions and six treatment sessions for 8 weeks. They will be assessed at baseline, and at 10 and 22 weeks after baseline. The primary outcome of this study is to evaluate whether the predetermined feasibility criteria for a full-scale trial are met. The potential primary outcome measures for a full-scale trial are the Social Communication Questionnaire and the Social Skills Improvement System. We will conduct a preliminary economic analysis. After the study has ended, a sample of 20 participants and their families will be invited to participate in semistructured interviews to explore the feasibility and acceptability of the study’s methods and intervention.

**Ethics and dissemination:**

Parents/carers will provide informed consent, and children will give assent, where appropriate. Care will be taken to avoid pressure or coercion to participate. Aftercare is available from the recruiting NHS Trust, and a phased withdrawal protocol will be followed if children become excessively attached to the robot. The results of the study will be disseminated to academic audiences and non-academic stakeholders, for example, families of children with ASD, support groups, clinicians and charities.

**Trial registration number:**

ISRCTN registry (ISRCTN14156001); Pre-results.

**Strengths and limitations of this study**

- This study will be the first randomised controlled trial of a robot-assisted social skills intervention for children with autism spectrum disorder.
- The study design, management and data analysis benefit from the input of experts by experience.
- Quantitative, qualitative and economic analyses will provide a comprehensive picture of feasibility.
- A definitive trial would be necessary to evaluate the effectiveness of the intervention.

## Introduction

Autism spectrum disorder (ASD) is a lifelong, neurodevelopmental condition that affects approximately 1 in 100 individuals in the UK.^1^ According to the *Diagnostic and Statistical Manual of Mental Disorders, Fifth Edition*, a diagnosis of ASD requires deficits to be present in social communication and interaction and restricted, repetitive patterns of behaviour, interests or activities.^2^ Given the profile of difficulties associated with ASD, individuals may experience challenges with independent living, self-care, developing meaningful relationships and educational and employment prospects.^3 4^ Furthermore, ASD does not just affect the health of the individual; parenting a child with ASD is a strong factor in parental anxiety and depression.^5 6^

Any intervention should focus on developing children’s social skills as it has been shown that social competence is a predictor of long-term outcomes for individuals with ASD.^7^ Furthermore, it is well established that early intervention yields best outcomes,^4 8 9^ so an intervention delivered soon after diagnosis affords the child the best opportunity to develop the basic skills required to fuel social competence.

The National Institute for Health and Care Excellence (NICE) guidelines indicate that children should be offered psychosocial intervention as a first-line treatment.^10^ One specific avenue of research has explored the use of such an intervention using social robots to support the social and emotional development of children with ASD. Robots have been shown to be effective social mediators, encouraging and facilitating social behaviour between children with ASD and developing their social skills.^11–14^

Kinesics and Synchronisation in Personal Assistant Robotics (Kaspar) is a minimally expressive humanoid robot. People’s social behaviour can be very subtle and can appear widely unpredictable to a child with ASD. Kaspar’s face has been depersonalised by reducing the detail so that the facial features are easily recognisable,^15^ therefore aiming to help children with ASD to identify and understand facial expressions and social interactions.^16^

Psychosocial interventions for ASD should include play-based strategies to increase joint attention, engagement and reciprocal communication.^10^ The games or scenarios that children can play with Kaspar have been designed so that they each contain elements of joint attention, imitation, turn-taking, cause and effect and collaboration. Evidence from case studies has shown promising results from using Kaspar with children with ASD. Teachers have reported improvements in children’s behaviour.^17^ Collaborative and play skills among children with ASD have shown improvement following sessions with Kaspar and another child,^16^ and carers are positive about the effects of the intervention on their child.^15^

The use of robot-assisted therapy with children with ASD is a rapidly growing area.^18–22^ However, although previous research suggests numerous educational and therapeutic benefits for children, much of what is known about the use of robots in interventions has been gleaned from single case reports. Virtually all peer-reviewed studies in this field have been preliminary and exploratory,^21^ but research demonstrating the effectiveness of social skills interventions should use rigorous research designs.^23^ As such, there is a need for studies to use group research designs, especially true experimental designs such as randomised controlled trials (RCTs) in order to unearth the usefulness of robot-mediated interaction for children with ASD.

### Aims and objectives

The present research aims to conduct a feasibility study of an RCT exploring the effectiveness of a humanoid robot to support social skills development in children with ASD. This feasibility study has a single-blind, randomised design, which will compare the Kaspar group (KG) to a group who have the same interaction with a therapist only (TOG), thereby exploring any specific effects of using a robot to facilitate the delivery of a social skills intervention. This article reports the protocol (v3.0, 24 February 2017) for the ‘Kaspar RCT’ study and follows SPIRIT (Standard Protocol Items: Recommendations for Interventional Trials) reporting guidelines.^24^

The study objectives are to assess the feasibility and acceptability of:

1. recruiting children with ASD to a randomised study and exploring rates of attrition
2. data collection for the outcome measures
3. estimating cost-effectiveness in a definitive trial
4. integrating a social skills intervention within the routine activity of an NHS Trust’s ASD diagnostic clinic
5. the study’s procedures and intervention among parents and children with ASD.

The following criteria will have to be met, or robust evidence will have to be presented to amend the study design, to proceed to a full-scale RCT:

1. More than 40% recruitment rate;
2. Less than 35% attrition;
3. Completion of at least 80% of the questionnaires;
4. Any issues with study design identified in the feasibility study can be addressed;
5. Good acceptability of the intervention among patients and their families as indicated in qualitative feedback;
6. Positive feedback from clinical staff about scheduling clinics;
7. The 80% CI of the effect size between the groups on a potential primary outcome measure excludes zero.

## Methods and analysis

### Design and setting

A single-blind feasibility RCT with two parallel groups based at a single NHS Trust in England (Hertfordshire Community NHS Trust) will be conducted for 24 months. Children will be randomised to receive an intervention with Kaspar and a therapist (KG), or the same intervention but with a TOG.

This study is a mixed design with between-group and within-participant comparisons to explore the feasibility of delivering a large-scale RCT.

### Participants

Forty children will be recruited through clinics in Hertfordshire Community NHS Trust that provide assessment and support to children with ASD, for example, the Communication Disorders Assessment Clinic (CDAC) in Watford to which approximately 1000 children a year are referred for a potential ASD diagnosis. The clinical team will screen children from their existing records and identify eligible children. Children who have been diagnosed in the last 12 months will be identified in the first instance with this time period being extended if necessary for recruitment.

### Inclusion/exclusion criteria

Inclusion criteria

- Aged 5–10 years;
- Diagnosis of an ASD confirmed with the Autism Diagnostic Observation Schedule^25^ and/or the Autism Diagnostic Interview^26^ within the last 12 months;
- Able to understand or fluently speak English;
- IQ >70.

Exclusion criteria

- Currently in receipt of a privately delivered social communication intervention (ie, not part of NHS or education usual care);
- The child is non-English speaking or if non-verbal, unable to understand English;
- Carer who is completing the questionnaire is unable to do so in English.

### Recruitment procedure

**Figure 1:**
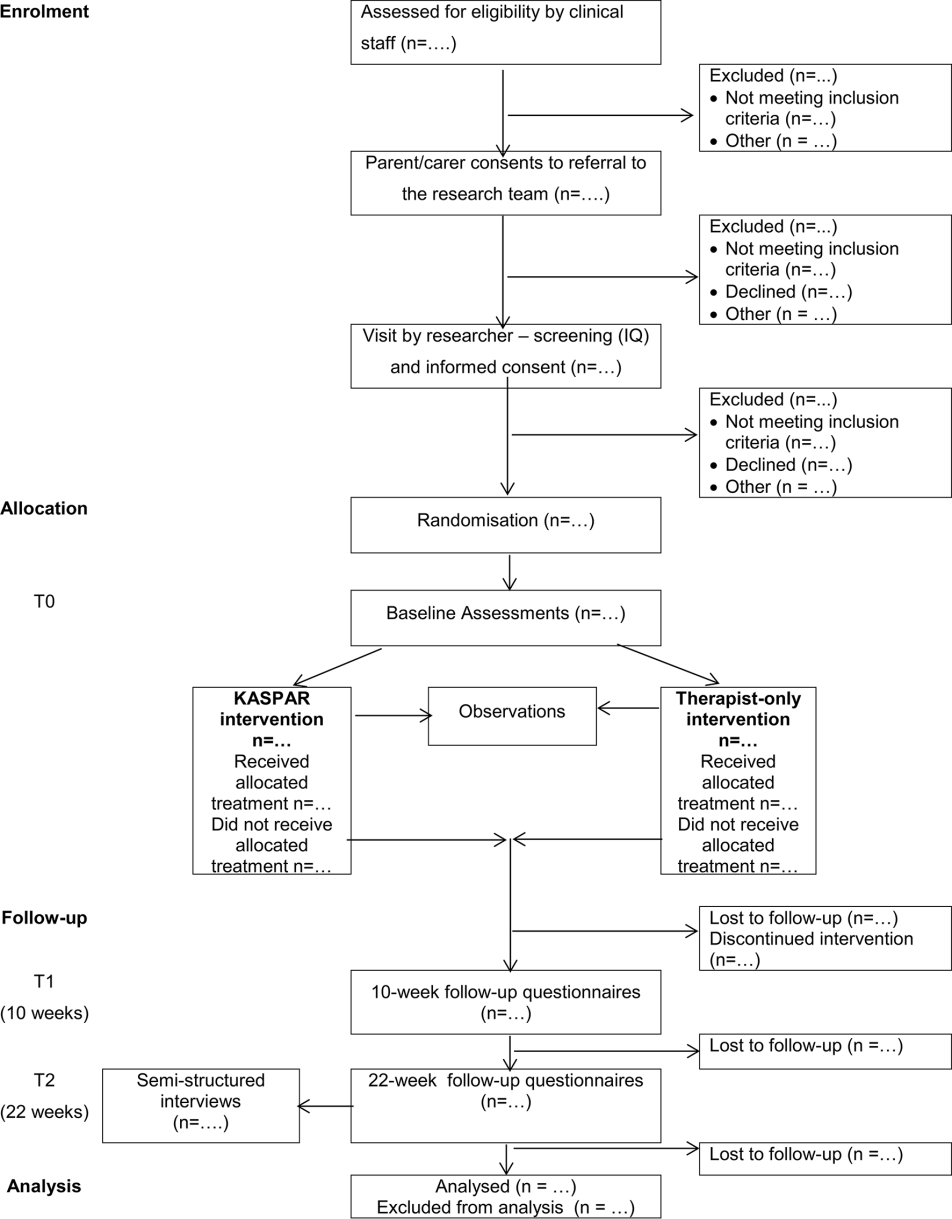
Study flow chart. Kaspar, Kinesics and Synchronisation in Personal Assistant Robotics.

Figure 1 shows the flow of participants through the study. Parents/carers of children meeting the inclusion criteria will be given details of the study at a routine clinic appointment or over the phone by a member of the clinical team, and they will be invited to complete a consent form to refer them to the research team for further information about the study. They will also have the opportunity to complete an ‘opt-out’ form if they would prefer not to be contacted again about the study. Information sheets designed for parents and children will be used. For all screened children, the clinical team will note down whether they were referred to the research team, if they were not eligible (and why) or if they declined (and why, if a reason is given). For those children who are referred to the research team, the parent/carer will be contacted approximately 2 weeks later and will be invited to a meeting with a member of the research team. This will be at a mutually convenient time and place, for example, the family’s home, the clinic or the University of Hertfordshire. A researcher will explain the project using the parent and child information sheets, and if the parent/carer would like to take part, then they will take consent from the parent/carer and assent from the child, where appropriate. Parents/carers who consent to study participation will also be asked if they consent to be recontacted at a later stage about participating in a semistructured interview.

The researcher will carry out an IQ test with the child to ensure that they meet the eligibility criteria of an IQ of at least 70. The Universal Non-verbal Intelligence Test^27^ will be used as it has completely non-verbal instructions and is for use with children between 5 and 21 years.

All members of the clinical and research team involved in recruitment will be trained in good clinical practice (GCP). The child’s GP and school will be informed about the study, if the parent/carer consents to this.

#### Randomisation

After the initial visit from the research team, the child will be randomised to either the KG or TOG. Individual randomisation will be performed on the online study database on Qualtrics by the trial coordinator, and the outcome will be shared with the therapist.

Families will be contacted when appointments for the intervention sessions are available. Parents/carers will not be informed which group their child has been randomised to at this point. They will be informed after the baseline assessment has been conducted (ie, before the intervention begins) to ensure that any disappointment at group allocation does not affect the baseline completion of the study questionnaires.

The trial coordinator, therapist and participants are unable to be blinded, but the research assistant who will collect data at T0 (baseline), T1 (10 weeks postbaseline) and T2 (22 weeks postbaseline) will be blinded. In order to maximise the chance that the research assistant remains blind to allocation, parents/carers will be asked not to share with the research assistant which group they have been allocated to. Any breach in blinding will be reported to the trial steering group (TSG) and will inform the design of a definitive RCT.

### Kaspar and therapist-only interventions

At the start of the intervention period, each child will receive two 15 min sessions with Kaspar and the therapist (KG) or with the therapist (TOG), depending on their group allocation. The purpose of these sessions will be to familiarise the children with Kaspar and/or the therapist. The intervention itself will consist of six weekly sessions with Kaspar and the therapist (KG), or with the therapist (TOG). KG children will be encouraged to interact with the therapist in order to play with Kaspar. The interactions will last for up to 20 min. A research nurse will act as the therapist and will receive full training in the social skills intervention and how to use Kaspar.

The weekly intervention sessions will involve the child playing a series of games with the therapist and/or Kaspar. The same games will be played in both groups, but the KG will play these games with the therapist and Kaspar and the TOG will play these games with the therapist. The games to be played have been designed so that they contain elements of joint attention, imitation, turn-taking, cause and effect and collaboration, and the therapist can select games based on the child’s responses in the session. It is important that any intervention to be used with children with ASD can be adapted for the individual child, as each child’s interests, preferences and capabilities may vary significantly.^20^

The interventions will be run as would normally be done in an ASD diagnostic clinic. Therefore, a list of participants for each group will be kept until a minimum number of participants (approximately six) have accrued for the therapist to run clinic sessions. Parents/carers will then be contacted and informed of available dates. Both interventions will be delivered in a clinic in Hertfordshire Community NHS Trust with a one-way mirror, which will allow parents to observe the intervention. The intervention sessions will also be video-recorded (depending on parental consent) so that the therapist can review the sessions and observations can be coded. The therapist will complete an intervention record form for each child’s familiarisation and therapy session.

Once their data collection has been completed, each child in the TOG will be given the opportunity to receive the intervention in the same way as the KG.

### Assessments

Each participant will be assessed through screening measures and three questionnaires at baseline, 10 weeks later and 22 weeks later in face-to-face research visits with a trained research assistant at a convenient location for the families (eg, their home/the clinic/the university). Observational data will also be collected during the intervention. A subgroup of participants will be invited to take part in a semistructured interview in order to glean their perspectives on the intervention and study process.

#### Baseline and follow-up questionnaires

Shortly before their first clinic appointment, the research assistant will contact the parent/carer to arrange the baseline assessment at a mutually convenient location. This is so the parent/carer can ask questions and also to ensure that the questionnaires are completed. This meeting will take place at a location that is most suitable for the participant (their home/clinic/university). After this visit, the parent/carer will be informed about which group their child has been allocated to by the trial coordinator or therapist.

Within 2 weeks of the end of the intervention (approximately 10 weeks after the baseline assessment), the child’s parents/carers will be contacted to arrange a visit to complete the questionnaires again.

Three months after the end of the intervention (approximately 22 weeks after the baseline assessment), parents/carers will again be contacted to arrange the final data collection visit.

**Figure 2:**
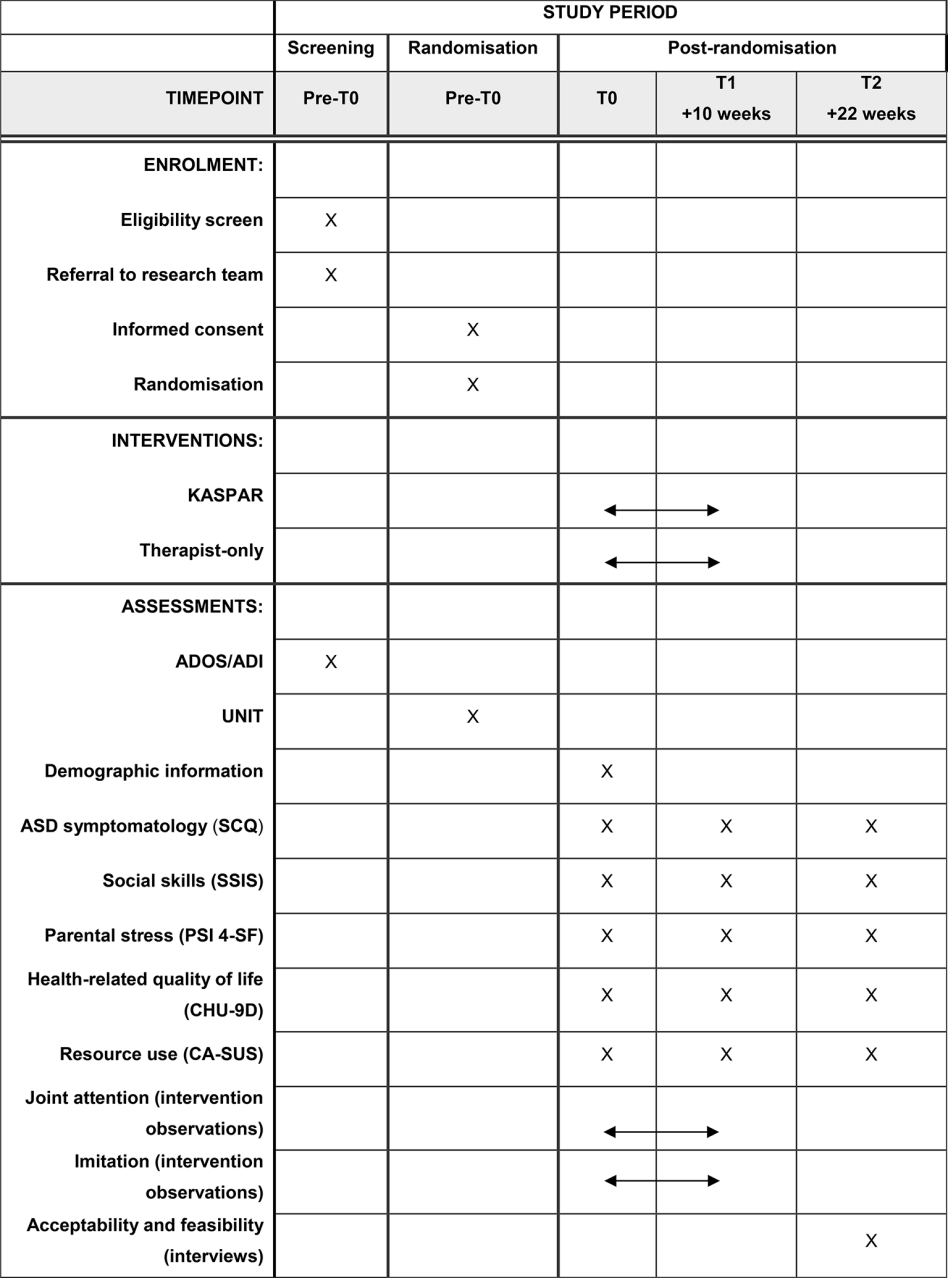
Study schedule. ADI, Autism Diagnostic Interview; ADOS, Autism Diagnostic Observation Schedule; CA-SUS, Child and Adolescent Service Use Schedule; CHU-9D, Child Health Utility 9D; Kaspar, Kinesics and Synchronisation in Personal Assistant Robotics; PSI-4-SF, Parenting Stress Index (Fourth Edition, Short Form); SCQ, Social Communication Questionnaire; UNIT, Universal Non-verbal Intelligence Test.

The questionnaires to be used in the baseline and follow-up assessments can be seen in figure 2. The measures selected have been widely used in the target population and are straightforward to complete. The potential primary outcome measures for the full-scale trial are the Social Communication Questionnaire (SCQ)^28^ and the Social Skills Improvement System (SSIS).^29^ The SCQ is a parental questionnaire assessing child behaviour associated with ASD, and the SSIS is a parental questionnaire assessing children’s social skills and problem behaviours. The Parenting Stress Index (Fourth Edition, Short Form) will likely be a secondary outcome measure in the full-scale trial and is a self-report questionnaire with three subscales: parental distress, parent–child dysfunction interaction and difficult child.^30^

The economic analysis will use the Child Health Utility 9D (CHU-9D)^31^ and an amended version of the Child and Adolescent Service Use Schedule (CA-SUS).^32^ The CHU-9D is a health-related quality of life questionnaire. The self-report version will be used with the child where possible, and the proxy version will be used for parental completion where the child is deemed as not able to complete the questionnaire. The CA-SUS is a parent/carer self-report questionnaire, which will collect information about the child’s use of NHS, personal social services (PSS) and education costs, as well as parent/carer costs.

#### Intervention observations

All intervention sessions will be video-recorded with consent from parents. Each participant’s first and final intervention session, that is, not the familiarisation sessions, will be coded for frequency and duration of joint attention and imitation using the observer system. To ensure reliability, 20% of the recorded intervention sessions will be independently coded by two researchers, and Cohen’s kappa will be computed.

#### Semistructured interviews

Interviews with parents/carers and children will be conducted by a trained researcher and with a sample of 10 participants from each group. Purposeful sampling will be used to ensure a diverse range of views are represented, for example, a range of ages, ability levels and dates of entry into the study. When the research assistant arranges the 22 -week follow-up, they will also ask if the selected parents/carers would be happy to take part in an interview, which would occur shortly after the visit to complete the questionnaires. Information sheets and consent/assent forms will be used to explain the purpose of the interviews and what would be involved.

The interviews will take place at a location that is most suitable for the participant (their home/clinic/University) and will be audio-recorded. A semistructured interview topic guide will be followed and will address the feasibility and acceptability of the recruitment procedures, questionnaire completion and intervention.

Towards the end of the study, health professionals will also take part in an audio-recorded semistructured interview to explore the feasibility of the trial design and intervention. Key staff members will be contacted and provided with an information sheet, and if they choose to take part, then they will be asked to complete a consent form.

### Study termination

The final questionnaire assessment point will be at week 22, and for those who are taking part in interviews, these will be arranged to take place shortly after the questionnaire completion. After this, children in the KG will resume care as usual. Children in the TOG will be offered sessions with Kaspar at the end of the study, which will follow the same format as the sessions received by the KG.

If a child withdraws from the study at any point, then they will be referred to usual care.

### Data analysis

The data analysis will enable the feasibility criteria to be addressed to inform the decision about recommending a definitive trial and will explore the following outcomes:

- The demographical and clinical characteristics of those who take part in the study;
- Recruitment rates (per month);
- Rate of attrition of participants in each arm of the study;
- Completion rates of the outcome measures;
- The suitability of outcome measures for the definitive trial;
- The feasibility of collecting resource use and quality of life data;
- The acceptability of the intervention to clinicians and parents;
- The practicality of running the clinics where the intervention is delivered in the NHS setting.

Data will be entered to the online study database, and this will be primarily undertaken by the research assistant, with a random 10% of questionnaires being checked by another member of the research team.

Stata will be used to conduct the statistical analysis, which will seek to evaluate the feasibility of undertaking a large-scale trial. The analysis will primarily be descriptive, aimed at estimating the parameters (mean, SD and proportions) required to design a definitive trial. Thus, the analysis will tabulate the data required to inform the decision whether to proceed: number of patients identified, number and proportion of patients recruited, proportion of patients completing the intervention and proportion of questionnaires completed.

Regarding potential outcome measures for the main study, the observed effect sizes of group differences will be observed at the two follow-ups, and the extent to which these outcomes indicate an effect size >0 will be calculated.^33^ The analysis will also consider which of the potential primary outcomes (SSIS and SCQ) is likely to be more sensitive to the intervention. These data will be used to inform sample size estimation for a future definitive trial. The findings from the observational coding will be reported, and the ease and reliability of this coding will inform the design of a full-scale trial.

Estimation of cost-effectiveness, within a health-technology assessment, is an iterative process.^34^ Levels of resource use and quality of life will be monitored to inform the decision as to how costs and benefits should be measured as part of a definitive study. Associated unit costs^35^ will be assigned to all items of NHS, PSS and education resource use indicated on the CA-SUS. Responses to the CHU-9D can be converted into a utility score (a scale where death is equal to 0 and full health 1)^31^ and, in turn, quality-adjusted life year scores.^36^

Interview data will be analysed using thematic analysis^37^ in NVivo, a qualitative data analysis software programme. The emerging themes will be discussed with the patient and public involvement (PPI) representatives who will receive basic training in the methodology run by a member of the research team with qualitative expertise.

### Sample size and feasibility of recruitment

As a feasibility study, the sample size is estimated following Cocks and Torgerson.^33^ The outcome measures intended for the definitive trial will be determined by this study, and the primary outcome measure will either be the SCQ or the SSIS. We estimate the power for this study assuming the effect size for the full trial will be at least 0.3. Assuming α=0.05 and 1−β=0.8, the required sample size to be able to reject an effect size of 0 would be n=13 per arm. Assuming a sample size of n=20 per arm (n=40 in total) will allow for attrition of 35% in the study.

Children will be recruited through a clinic assessing children referred for communication disorders in Hertfordshire Community NHS Trust. In the first instance, we will work with the CDAC in Watford, as a preliminary audit confirmed that 125 participants meeting the inclusion criteria are diagnosed each year. Applying a refusal rate of 40% (as gleaned from our broad experience of research) would mean that 75 would take part. However, if recruitment rates are lower than expected, then children who have been diagnosed the previous year will be invited to participate and/or we will identify and approach additional clinics in Hertfordshire Community NHS Trust.

### Study management

The study started on 1 December 2016 and will finish on 30 November 2018. The study is cosponsored by Hertfordshire Community NHS Trust (lead sponsor) and University of Hertfordshire. The study is funded by the National Institute for Health Research (NIHR) who plays no role in the study design, conduct, analysis or report writing. The study was registered as a clinical trial with ISRCTN registry on 23 February 2017, and any changes to the protocol will be updated on the registry as necessary.

The TSG will comprise the protocol authors along with a representative from Hertfordshire Community NHS Trust Research and Development Office and the study research assistant. The TSG will meet quarterly and be responsible for monitoring the progress and conduct of the study, addressing key issues that may arise and reporting to the funder and the research ethics committee (REC). As this is a feasibility study involving low-risk interventions, it is considered that a data-monitoring committee is not needed. The TSG will have the responsibility to change the protocol and/or stop the trial at any point if needed, and to notify relevant parties, for example, the sponsors and REC.

The project advisory group will include core members of the study team and experts by experience. They will meet two times per year in person or by telephone conference. The meetings will be scheduled so that the output will inform the TSG meetings.

A core team involved in the day-to-day running of the study will form the trial management group to ensure all practical aspects of the trial are progressing well and to identify potential issues as early as possible. They will meet on a monthly basis in person, by telephone conference or through email discussion, where appropriate.

#### Adverse events

From previous studies with Kaspar and other psychosocial interventions with children with ASD, the likelihood of a (serious) adverse event or reaction occurring is low. However, if these are suspected to have occurred, they will be recorded. The TSG and sponsors will be notified, and their guidance will be followed regarding further notification of other parties, for example, the REC.

### Data management

This study will be conducted in accordance with the Data Protection Act (1998) and the guidelines of the Declaration of Helsinki (1964; updated Tokyo, 2004).

Data management will be coordinated from the University of Hertfordshire. Access to patient-identifiable data, for example, consent forms, will be restricted to authorised personnel. Only GCP-certified investigators approved by the TSG will be given access to the study data set; this will include members of the research team involved in the data analysis. A unique patient identification number (ie, a sequential number starting at K001) will be generated by the study web-based data entry system for each child that is screened by the clinical team. This will be used on all paper and electronic data. The electronic data management system being utilised for the purpose of the study, Qualtrics (http://www.qualtrics.com/), is a powerful online system that enables data to be securely uploaded and managed from different sites.

All participant data, associated files and hard copy questionnaires, including any that might identify participants, will be accessed only by the research team. Consent forms will be stored at Hertfordshire Community NHS Trust during the study and will be collected and stored at the University of Hertfordshire at the end of the study. Patient-identifiable data relating to the study will be deleted or destroyed within 6 months of the end of the study. Anonymised study data will be archived by the University of Hertfordshire for 5 years after study completion in line with standard research protocols. Investigators will not disclose patient data in any form to anyone not involved directly in the study. All electronic data will be stored on password-protected computers, and paper files will be stored in locked filing cabinets, both of which will be kept within locked offices. The video recordings of the intervention sessions will be transferred to an encrypted external hard drive and deleted from the device. The hard drive will be stored in a lockable cabinet in a locked office.

## Ethics and dissemination

### Ethical considerations

Ethical approval for this study was granted on 10 November 2016 by the Cambridge South REC (16/EE/0387). Initial Health Research Authority (HRA) approval was granted on 18 November 2016. Here, we report version 3.0 of the protocol, which received ethical and HRA approval on 15 March 2017. Any future amendments will be approved by the sponsors, HRA and REC as appropriate.

As parents/carers may be likely to be vulnerable to coercion around the time of diagnosis, the research team will not follow-up for 2 weeks after referral from the Hertfordshire Community NHS Trust clinic, at which time a researcher will go through the information and address any questions before taking consent. The participants are minors, so fully informed consent will be obtained from their parent/carer. A child-friendly written information sheet, a pictorial information sheet and a child assent form will also be used as deemed appropriate by the parents/carers and the study team. The information sheets and consent forms have been reviewed by parents of children with ASD and an adult with ASD.

The child will be told what is expected of them in the intervention sessions. The sessions will be paused or stopped if a child shows signs of becoming distressed. Their parent/carer will be watching through a one-way mirror so they will be able to comfort the child and escort them from the room if necessary.

If aftercare is required by a parent/carer or child, then appropriate support will be provided by drawing on the range of usual therapy, which is available in the NHS Trust, for example, play therapy. Where appropriate, parents/carers will also be signposted to the National Autistic Society for parent support.

To date, studies using Kaspar have not experienced any difficulties in children becoming excessively attached to the robot. However, if this does occur, then we will follow a phased withdrawal protocol after discussion with their parents/carers, the therapist and a consultant paediatrician. Depending on clinic room availability and family preference, we will provide access to the robot at the University of Hertfordshire or Hertfordshire Community NHS Trust with a member of the study team. The nature of this will vary according to each child, but the principles of the phased withdrawal would be to reduce the length and frequency of sessions in an appropriate manner, for example, by explaining that Kaspar has other children to play with.

### Dissemination plan

The literature regarding the use of social robots in interventions with children with ASD is currently reliant on single case reports; thus, the evidence base requires methodologically robust research designs. Reliable research findings will lead to impact beyond the research community to practitioners in healthcare and education, and as such will facilitate access to wider patient benefit. Our dissemination plan has been designed with this in mind and was developed in close collaboration with PPI representatives.

Our dissemination and impact activities will include open-access academic publications in high-impact journals, presentations at academic and non-academic conferences, hosting a free entry public seminar about the study and producing briefings for parent groups, charities and NHS services. The timing and details for these dissemination activities will be discussed at TSG and project advisory group meetings.

The research team will write all articles submitted for peer-reviewed publications, and authorship inclusion and order will be guided by levels of contribution. All publication material will acknowledge the funding contribution from NIHR.

This is a shortened version of the full protocol, which is available on request from the corresponding author. Requests for access to the anonymised data and statistical code should also be addressed to the corresponding author.

## Discussion

This study will be the first to assess the feasibility of conducting an RCT to evaluate a robot-assisted social skills intervention for children with ASD. The results of this feasibility study will be used to decide whether to progress to a full-scale trial, and if so, what methodological issues may need to be addressed and changed.

NICE guidelines indicate that children should be offered a psychosocial intervention as a first-line treatment, and this should include play-based strategies to increase joint attention, engagement and reciprocal communication, which Kaspar is designed to do.^36^ A full-scale study will allow the evaluation of the effect of the Kaspar intervention soon after diagnosis. Offering an early intervention such as Kaspar has the potential to improve children’s social skills, to affect their ability to access other forms of intervention, for example, speech and language therapy, and to improve their long-term outcomes.

## Supplementary Material

Reviewer comments

Author's manuscript
