# Supplementary material for: Feasibility study of a randomised controlled trial to investigate the effectiveness of using a humanoid robot to improve the social skills of children with autism spectrum disorder (Kaspar RCT): a study protocol
Source: BMJ Open. 2017 Jun 22;7(6):e017376. doi: 10.1136/bmjopen-2017-017376 (PMC5726066; doi:10.1136/bmjopen-2017-017376)
Supplement: Author's manuscript [file bmjopen-2017-017376.draft_revisions.pdf]

PEER REVIEW HISTORY

BMJ Open publishes all reviews undertaken for accepted manuscripts. Reviewers are asked to complete a checklist review form (**http://bmjopen.bmj.com/site/about/resources/checklist.pdf**) and are provided with free text boxes to elaborate on their assessment. These free text comments are reproduced below.

ARTICLE DETAILS

**TITLE (PROVISIONAL)**	Feasibility study of a randomised controlled trial to investigate the effectiveness of using a humanoid robot to improve the social skills of children with an Autism Spectrum Disorder (KASPAR RCT): A Study Protocol
**AUTHORS**	Mengoni, Silvana; Irvine, Karen; Thakur, Deepshikha; Barton, Garry; Dautenhahn, Kerstin; Guldberg, Karen; Robins, Ben; Wellsted, David; Sharma, Shivani

This article was not externally reviewed at BMJ Open. Protocols that have been independently assessed prior to submission to BMJ Open are usually fast-tracked to publication on the grounds that further substantial changes will not be possible. This independent assessment will usually be external, independent review for both a substantial grant award from a non-commercial or government funder and ethics approval.
